# Cor triatriatum sinister with left anomalous pulmonary venous drainage to innominate vein: what to do with the vertical vein?

**DOI:** 10.1007/s11748-020-01533-w

**Published:** 2020-11-02

**Authors:** Keiichi Ishiwari, Koji Nomura, Yoshihiro Ko, Izumi Hamaya, Kodai Momoki, Tomomitsu Takagi

**Affiliations:** 1 Department of Cardiovascular Surgery, Saitama Children’s Medical Center, Saitama, Japan; 2 Department of Cardiology, Saitama Children’s Medical Center, Saitama, Japan

**Keywords:** Congenital heart disease, Cor triatriatum, Left anomalous pulmonary venous return, Vertical vein, Banding

## Abstract

We treated a surgical case of a 47-day-old male infant diagnosed with an unusual type of cor triatriatum sinister (CTS) with left anomalous pulmonary venous drainage to the innominate vein via a vertical vein. After preoperative hemodynamic assessment of pulmonary venous (PV) return, this patient underwent a resection of the fibromuscular membrane between the accessory and the true left atrial chambers, concomitant with vertical vein banding to facilitate a left PV return through a common pulmonary venous collector (CPVC). Catheterization three months after this surgery revealed no obstruction of the PV return to the mitral orifice as well as good growth of the CPVC as a left PV return pathway. The patient has been doing well on aspirin.

## Introduction

Cor triatriatum sinister (CTS) represents only 0.1% of all cardiac malformations [1]. Partial or total anomalous pulmonary venous return (PAPVR/TAPVR) is reported as a coexisting disease in 10–33% of patients with CTS [2], but left anomalous pulmonary venous drainage coexisting with CTS is less frequently described [3–7]. CTS with left anomalous pulmonary venous drainage may be hemodynamically similar to obstructive TAPVR when a common pulmonary venous collector (CPVC) is present, and surgical intervention may be urgently needed [3]. Here, we report a rare urgent surgical case of CTS associated with left anomalous pulmonary venous drainage.

## Case

A 47-day-old male infant was transferred to our center for evaluation of body weight loss.

**Fig. 1:**
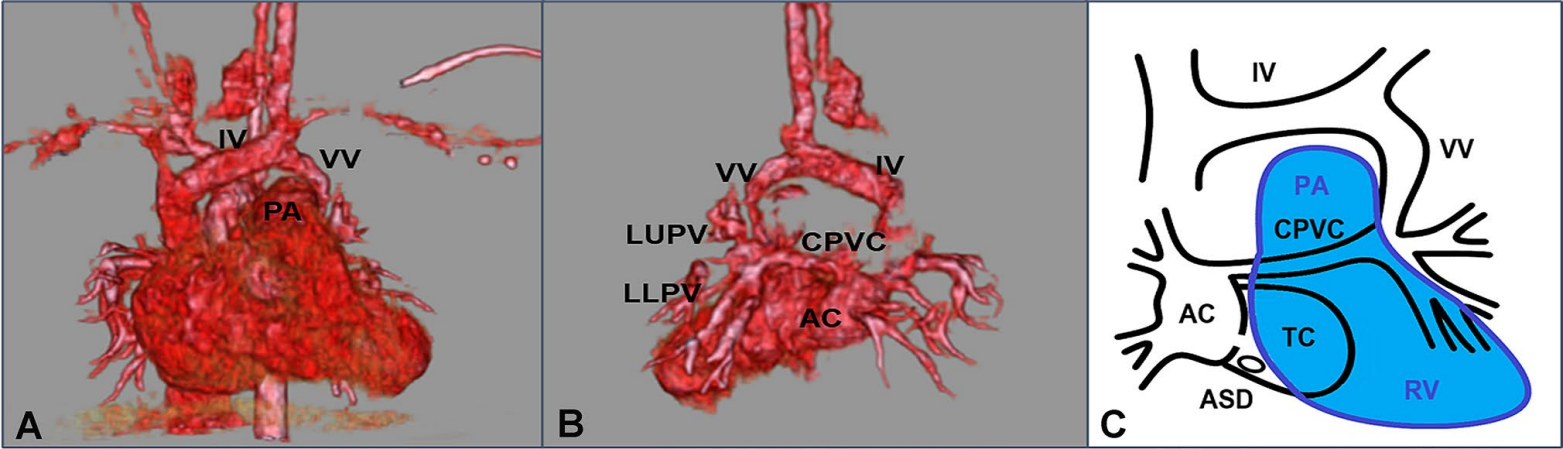
**a** Preoperative CT showing the left anomalous pulmonary venous drainage to the innominate vein (IV) and the vertical vein (VV) compressed from the dilated pulmonary artery (PA). **b** Preoperative CT showing the common pulmonary venous collector (CPVC) between the left upper/lower pulmonary vein (LUPV/LLPV) and the accessory left atrial chamber (AC). **c** Schema of the left anomalous pulmonary venous drainage. *ASD* atrial septal defect, *RV* right ventricle, *TC* true left atrial chamber

After admission, CT scan revealed CTS with left anomalous pulmonary venous drainage. The left pulmonary vein (PV) was draining into the innominate vein via the vertical vein (VV), as well as into the accessory left atrial (LA) chamber via a narrow common pulmonary venous collector (CPVC). He was diagnosed with CTS as a subtype of Lucas–Schmidt classification III A2 (Fig. 1).

**Fig. 2:**
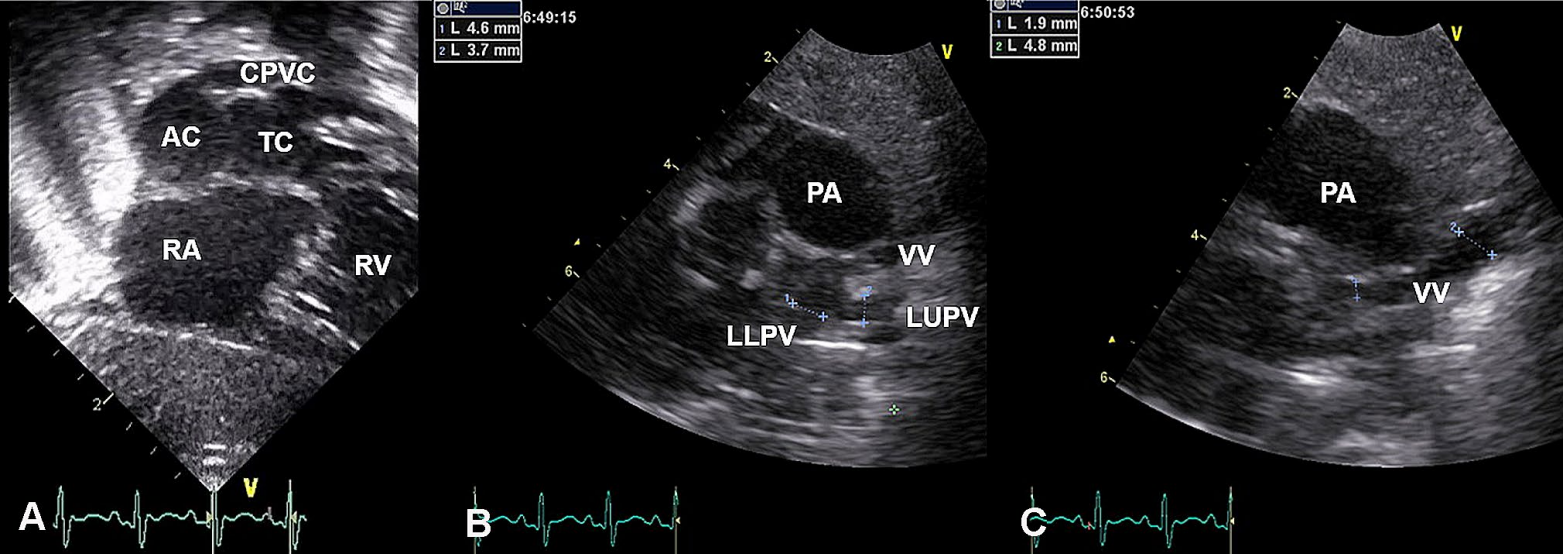
Preoperative transthoracic echocardiography. **a** The fibromuscular membrane dividing the left atrium and the common pulmonary venous collector (CPVC) connecting to the accessory left atrium (AC). **b** The left upper/lower pulmonary vein (LUPV/LLPV) draining into the vertical vein (VV). **c** The vertical vein compressed from the dilated pulmonary artery (PA). *RA* right atrium, *RV* right ventricle, *TC* true left atrial chamber

On hospital day 2, transthoracic echocardiography revealed a significantly dilated right portion of the heart and a severely obstructive fibromuscular membrane between the two LA chambers (mean pressure gradient 6.2 mmHg); this membrane contained a hole 2.9 mm in diameter. The atrial septal defect (ASD) was likewise restrictive with a diameter of about 2 mm (type A2 Lam classification [8]). The CPVC between the VV and the accessory LA chamber was as narrow as 2.9 mm in places. On the other hand, the VV was compressed from the dilated left pulmonary artery (PA) (Fig. 2).

**Fig. 3:**
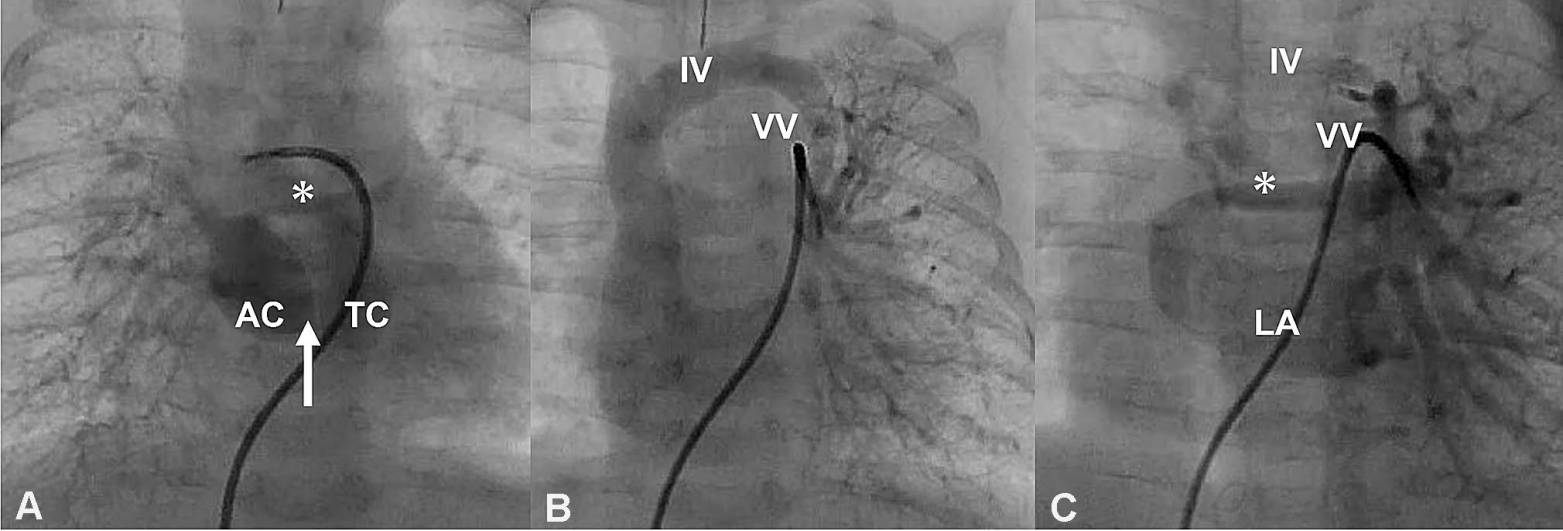
Pre/postoperative angiogram. **a** The small common pulmonary venous collector (asterisk) as seen on preoperative right pulmonary arteriogram. The fibromuscular membrane (white arrow) divided the left atrium into an accessory left atrial chamber (AC) and the true left atrial chamber (TC). **b** The vertical vein (VV) as seen on preoperative left pulmonary arteriogram. **c** Left pulmonary angiogram three months after surgery showing the thick common pulmonary venous collector (asterisk). *IV* innominate vein, *LA* left atrium

On hospital day 4, cardiac catheterization revealed super-systemic pulmonary hypertension with a pulmonary/systemic pressure ratio of 1.21. The mean pressure gradient between the two LA chambers was estimated at about 10 mmHg. The VV was not obstructive because left PCWP was 16 mmHg and mean innominate vein pressure was 14 mmHg (Table 1). Right PA angiogram showed that right pulmonary venous (PV) blood was partially flowing through the long narrow CPVC and then through the dilated VV (Fig. 3a). Left PA angiogram showed that left PV blood was flowing almost entirely into the VV (Fig. 3b).

**Table 1:** Hemodynamic data of pre/postoperative cardiac catheterization

Site	Pressure (mean) (mmHg)	
	Preoperative	Postoperative
Inn vein	(14)	(6)
SVC	(14)	(6)
IVC	(14)	(6)
RA	(13)	(6)
RV	60/–/EDP21	28/–/EDP1
Main PA	68/40(50)	26/12(17)
Right PA	67/39(49)	19/12(14)
Left PA	65/38(48)	20/12(16)
Right PCWP	(18)	(9)
Left PCWP	(16)	(7)
Left Up PV		(11)
LA	(8)	
AAo	56/35(44)	75/48(61)

*Inn vein* innominate vein, *SVC* superior vena cava, *IVC* inferior vena cava, *RA* right atrium, *RV* right ventricle, *PA* pulmonary artery, *PCWP* pulmonary capillary wedge pressure, *PV* pulmonary vein, *LA* left atrium, *AAo* ascending aorta, *EDP* end diastolic pressure

After cardiac catheterization, the patient underwent an urgent operation. Under standard cardiopulmonary bypass and cardioplegic arrest, both the right atrium and right-sided left atrium were opened. Three structures could be confirmed from the left atriotomy, ① right PV ② CPVC orifice, and ③ a small window to the true LA chamber, whereas the only structure of ASD was confirmed from the right atriotomy. The fibromuscular membrane between the two LA chambers was breached by a narrow communication less than 3 mm in diameter, which was completely resected. We did not choose to cut back the orifice of the CPVC because the CPVC had a long narrow shape rather than a localized stenosis in the opening of the LA. The ASD was repaired with a pericardial patch with a 4 mm fenestration to prevent pulmonary hypertension crisis. VV banding was applied by wrapping 2-0 silk around the VV, applying surgical clips, and adjusting the diameter of the VV to half its original value.

**Fig. 4:**
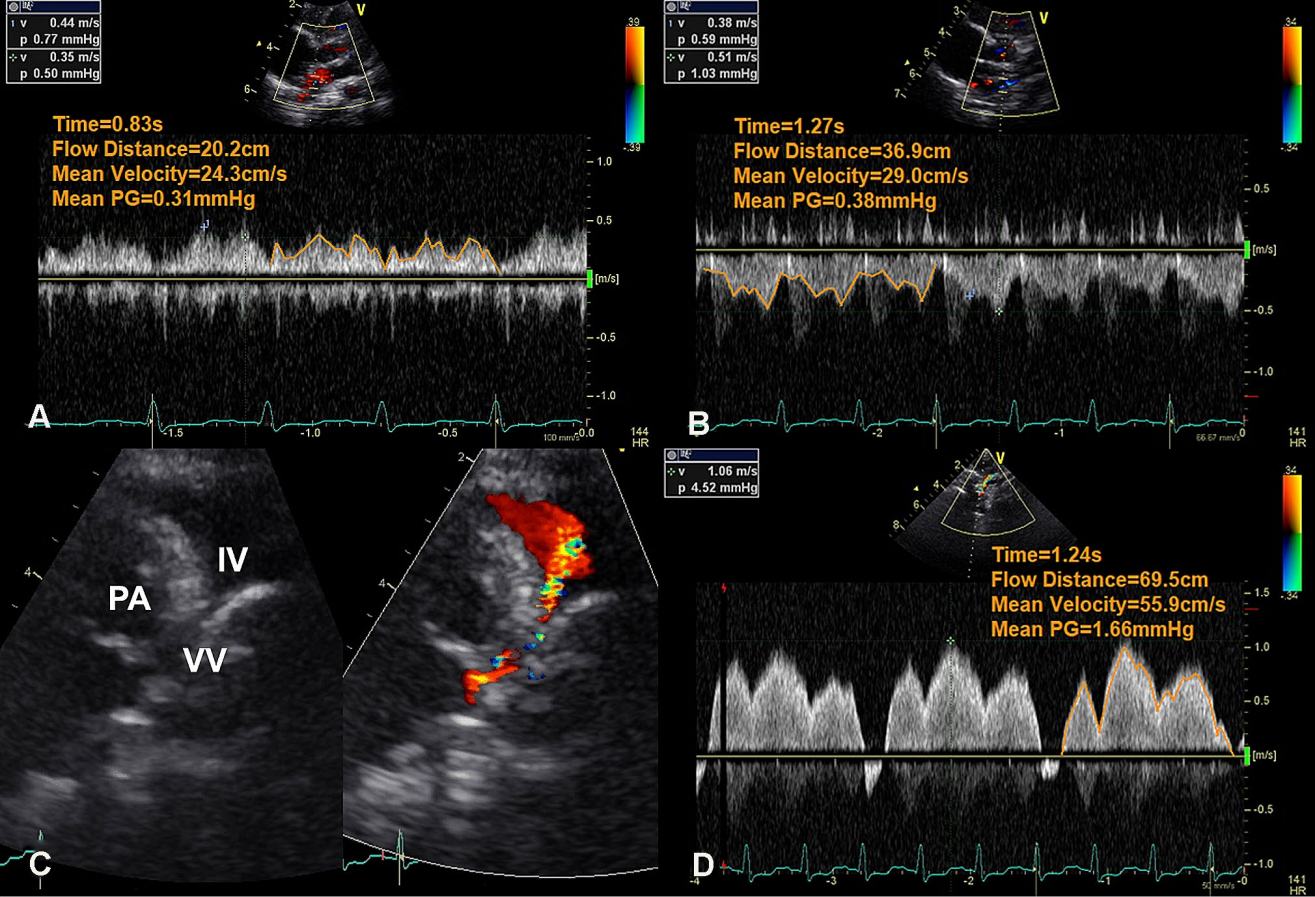
Postoperative transthoracic echocardiography. **a** Pulse Doppler of phasic flow between the two left atrial chambers. **b** Pulse Doppler of phasic flow in the common pulmonary venous collector. **c** The vertical vein (VV) and the turbulent flow in the banding site of the vertical vein as shown color Doppler. **d** Pulse Doppler of the phasic flow in the banding site of the vertical vein, varying with the cardiac cycle. *IV* innominate vein, *PA* pulmonary artery

The postoperative course was uneventful, with no episodes of pulmonary hypertension crisis. The patient was extubated on postoperative day (POD) 5. Postoperative transthoracic echocardiography showed low velocity with no obstruction in the two LA chambers or the CPVC. The mean pressure gradient in the VV is 1.7 mmHg (Fig. 4). He was discharged on POD 20.

Three months after the surgery, cardiac catheterization revealed that the blood flow from the left PV to the mitral orifice was smooth and unobstructed. The procedure also confirmed that the CPVC had grown, as had the left PV (Fig. 3). Main PA pressure dropped significantly to 26/11 mmHg (mean, 17 mmHg), and there was no significant difference in the left and right PA pressures. The mean pressure gradient at the banding of the VV is estimated at about 5 mmHg (Table 1).

## Discussion

In CTS, the LA is divided into two chambers by an obstructive fibromuscular membrane. The accessory LA chamber receives the pulmonary veins, while the true LA chamber contains the mitral valve and LA appendage. CTS is associated with other congenital cardiac anomalies [2, 9], most commonly PV return abnormalities. The basic surgical management for CTS is the resection of the fibromuscular membrane inside the LA, accompanied if necessarily by surgical intervention for concomitant cardiac malformations. In CTS associated with left anomalous pulmonary venous drainage, the necessity of surgical intervention on the VV varies among patients depending on anatomical and hemodynamic evaluation [3–7].

The first successful surgical repair of CTS associated with left PAPVR was reported in 1977 by Jennings et al. [5]. Their patient was a 10-week-old infant with an obstructive subtotal cor triatriatum with left PAPVR (Lucas–Schmidt classification III A2). The accessory LA chamber received only the right pulmonary veins, whereas the left pulmonary veins all returned to the innominate vein via the VV. The VV was divided and anastomosed with a 6-mm opening to the LA appendage. Nevertheless, this case exhibited left atelectasis after surgery, suggesting left pulmonary congestion. Since then, CTS has been reported to be associated with PV stenosis both before and after repair in more than 10% of cases [2]. Even if short-term patency is not an issue, it is worth being concerned about long-term patency in the VV and LA anastomosis, especially for small children.

Galoin-Bertail and Gouton reported a 14-year-old girl with CTS with left anomalous pulmonary venous drainage to the innominate vein in whom the left inferior PV connection to the accessory LA chamber was extremely similar to that in our case [4]. In that case, however, they resected the fibromuscular membrane without involving the PV because of the wide communication between the left PV and the accessory LA chamber.

In our case, the left upper and lower PV flow mainly drained into the VV and from there into the innominate vein, while the left PV blood flow did not drain into the narrow CPVC that connected the accessory LA chamber and the left PV because of the pressure gradient. In addition to resection of the fibromuscular membrane, we had three surgical options: ① VV ligation, ② direct anastomosis of the left PV and the LA appendage, and ③VV banding. After thorough hemodynamic evaluation, we selected VV banding because the CPVC was too small to receive the whole volume of blood from the left lung. Adequate growth of the CPVC was essential to avoid left PV stenosis, and we expected VV banding to promote growth of the CPVC. In addition, it is very difficult to achieve an adequately sized anastomosis between the left PV and the LA in early infancy. Therefore, VV banding was the procedure of choice for two advantageous reasons: (1) It promoted CPVC growth, and (2) it served as an escape route for the left PV blood flow, preventing left pulmonary congestion until the CPVC reached an adequate size. In fact, postoperative catheterization showed an unexpected development of collateral circulation from the accessory hemiazygos vein to the superior vena cava. This suggests that left PV pressure was high during the postoperative acute phase, and that VV ligation might have caused left pulmonary congestion.

Tightness of the band was another concern. In this infant, the VV had to be downsized by nearly half its diameter. Postoperative catheterization showed the mean pressure gradient of 5 mmHg in the VV, which is somewhat overestimated. On the other hand, postoperative echocardiography showed pulsatile antegrade blood flow at the banding site of the VV and a mean pressure gradient of 1.7 mmHg, which is considered an accurate assessment. The growth of the CPVC was confirmed by cardiac catheterization 3 months after surgery, suggesting “effective banding”, which facilitates PV return to CPVC without left pulmonary congestion during the postoperative acute phase. A strong band of less than half the original diameter could have led to a VV occlusion, yet a loose band of more than half the original diameter would not have promoted CPVC growth. In future cases, although the CPVC might not grow, the anastomosis of the dilated VV and the LA could lead to longer patency, especially in older children.

The catheterization 3 months after the surgery revealed no difference between the left and right PA pressure, suggesting no significant difference between the left and right PV return. However, the long-term effects of VV banding on pulmonary arteries and veins are unknown, and pulmonary perfusion scintigraphy should be performed in the future. The small left-to-right shunt through the banded VV will be closed surgically or interventionally if needed.

## Conclusion

We treated a case of cor triatriatum sinister similar to Lucas–Schmidt III A2 with left anomalous pulmonary venous drainage. Since the duration of patency in surgical repair for left anomalous pulmonary venous drainage in early infancy is uncertain, vertical vein banding should be the procedure of choice to promote growth of the common pulmonary venous collector.
